# Plant diversity drives soil carbon sequestration: evidence from 150 years of vegetation restoration in the temperate zone

**DOI:** 10.3389/fpls.2023.1191704

**Published:** 2023-06-06

**Authors:** Qilong Tian, Xiaoping Zhang, Haijie Yi, Yangyang Li, Xiaoming Xu, Jie He, Liang He

**Affiliations:** ^1^ The Research Center of Soil and Water Conservation and Ecological Environment, Chinese Academy of Sciences and Ministry of Education, Yangling, Shaanxi, China; ^2^ Institute of Soil and Water Conservation, Chinese Academy of Sciences and Ministry of Water Resources, Yangling, Shaanxi, China; ^3^ University of Chinese Academy of Sciences, Beijing, China; ^4^ Institute of Soil and Water Conservation, Northwest A&E University, Yangling, China; ^5^ College of Urban, Rural Planning and Architectural Engineering, Shangluo University, Shangluo, China

**Keywords:** biodiversity, carbon sink, vegetation succession, forest plantation, soil organic carbon

## Abstract

Large-scale afforestation is considered a natural way to address climate challenges (e.g., the greenhouse effect). However, there is a paucity of evidence linking plant diversity to soil carbon sequestration pathways during long-term natural restoration of temperate vegetation. In particular, the carbon sequestration mechanisms and functions of woody plants require further study. Therefore, we conducted a comparative study of plant diversity and soil carbon sequestration characteristics during 150 years of natural vegetation restoration in the temperate zone to provide a comprehensive assessment of the effects of long-term natural vegetation restoration processes on soil organic carbon stocks. The results suggested positive effects of woody plant diversity on carbon sequestration. In addition, fine root biomass and deadfall accumulation were significantly positively correlated with soil organic carbon stocks, and carbon was stored in large grain size aggregates (1–5 mm). Meanwhile, the diversity of Fabaceae and Rosaceae was observed to be important for soil organic carbon accumulation, and the carbon sequestration function of shrubs should not be neglected during vegetation restoration. Finally, we identified three plants that showed high potential for carbon sequestration: *Lespedeza bicolor*, *Sophora davidii*, and *Cotoneaster multiflorus*, which should be considered for inclusion in the construction of local artificial vegetation. Among them, *L. bicolor* is probably the best choice.

## 1 Introduction

The loss of plant diversity is a major challenge faced by humans in maintaining the stability and functional sustainability of ecosystems (Hautier et al., 2015; Hua et al., 2022). Anthropogenic activity such as deforestation and landuse changes causes 30% reduction in C (carbon) stock (Bargali et al., 2018; Awasthi et al., 2022a Manral et al., 2020; Manral et al., 2022; Bisht et al., 2023). Population pressure, agricultural expansion/intensification and development of infrastructure have been considered as major threats to biodiversity (Davidar et al., 2010; Baboo et al., 2017; Bargali et al., 2019; Bargali et al., 2022; Bisht et al., 2022) which causes an increase in CO_2_ in the atmosphere. Carbon sequestration is a key ecosystem function influenced by plant diversity (Bartelt-Ryser et al., 2005; Isbell et al., 2018). Forests are an important component of the global carbon cycle as they store 70–90% of terrestrial above and belowground biomass and are a major carbon sink, which is strongly linked to the diversity of plants in different forest types (Aponte et al., 2020; Besnard et al., 2021). There is much uncertainty about whether tropical forests are carbon sinks or sources, while temperate forests are known to play a significant role as terrestrial carbon sinks (Clark, 2004; Yang et al., 2022).

In recent decades, the increasing emissions of CO_2_-based greenhouse gases in the atmosphere (CO_2_ emissions have increased from 280 ppm in the pre-industrial era to 400 ppm today) have led to a series of environmental problems, such as global warming, sea level rise, and increase in extreme weather events, which seriously threaten the sustainable development of natural ecosystems and socio-economic systems (Zhang et al., 2011; Ramachandra and Bharath, 2020). Vegetation restoration is often used to increase soil organic carbon (SOC) storage and sequestration to reduce CO_2_ emissions and restore ecosystem functions (Zhao et al., 2015; Pandey et al., 2023; Shahi et al., 2023). Such restoration promotes the accumulation of SOC, making SOC the largest component of the terrestrial SOC pool, which is consequently two to three times greater than the vegetation carbon pool and plays a crucial role in regulating global warming (Davidson et al., 2000; Lal, 2004). In forest ecosystems, soil is an important participant and carrier of matter and energy. It provides essential mineral nutrients and water for plant growth. It can be a carbon sink or source, and has become a hot topic in the discussion on global climate change (Deng et al., 2013). Plant-soil interactions are important intrinsic drivers of ecosystem evolution. Many studies have focused on plant dynamics during vegetation succession (Teixeira et al., 2020; Prach et al., 2021), the physical and chemical properties of soils (Gu et al., 2019; Zhang et al., 2021), microbial turnover and change (Gavazov et al., 2022; Hu et al., 2022), and the effect of vegetation type changes caused by natural restoration and afforestation on soil carbon sequestration capacity (Liu et al., 2015; Yang et al., 2019a; Wang et al., 2020; Yan et al., 2020; Augusto and Boča, 2022). However, these factors are usually considered to influence soil carbon accumulation individually or independently. Currently, experimental evidence from grasslands and subtropical and tropical studies indicates that plant diversity increases biomass production and SOC storage (Craven et al., 2018; Chai et al., 2019; Yang et al., 2019b; Osuri et al., 2020; Yu et al., 2020). It has also been recognized that increasing plant diversity may improve plant productivity through complementary effects of ecological niches (Duffy et al., 2017). This consequently promotes plant input of carbon-containing material into the soil and increases SOC accumulation (Chen et al., 2020). However, there is a paucity of evidence linking vegetation diversity to soil carbon sequestration pathways on a broad scale during long-term natural recovery of temperate vegetation. Furthermore, tree-based ecosystems are critical for climate change mitigation; however, the mechanisms of carbon sequestration by woody plants are poorly understood, and there are also few reports on the plants that participate in carbon sequestration.

To elucidate these processes, in the present study, we investigated the influence of plant diversity on soil carbon sequestration by examining the natural recovery process of vegetation in the temperate zone for up to 150 years, considering sample sites, vegetation composition at different time periods, plant diversity, fine-root biomass, litter accumulation, soil aggregates, and SOC characteristics. Representative stand types and major species assemblages were selected and combined with partial least squares path modelling (PLS-PM) to quantify the contribution of plant diversity to soil carbon storage and evaluate the direct and indirect effects of the diversity of key plant families on carbon sequestration pathways. The aim of this study was to answer the following questions: 1) What are the characteristics of changes in vegetation and SOC during long-term vegetation recovery in the temperate zone? 2) How does plant diversity drive soil carbon sequestration? 3) What are the plants associated with soil carbon sequestration? Understanding how they are connected and how they work is important for managing ecosystem carbon pools, restoring vegetation to a near-natural state, and improving ecological management, which is important for addressing global climate change.

## 2 Materials and methods

### 2.1 Study area

**Figure 1:**
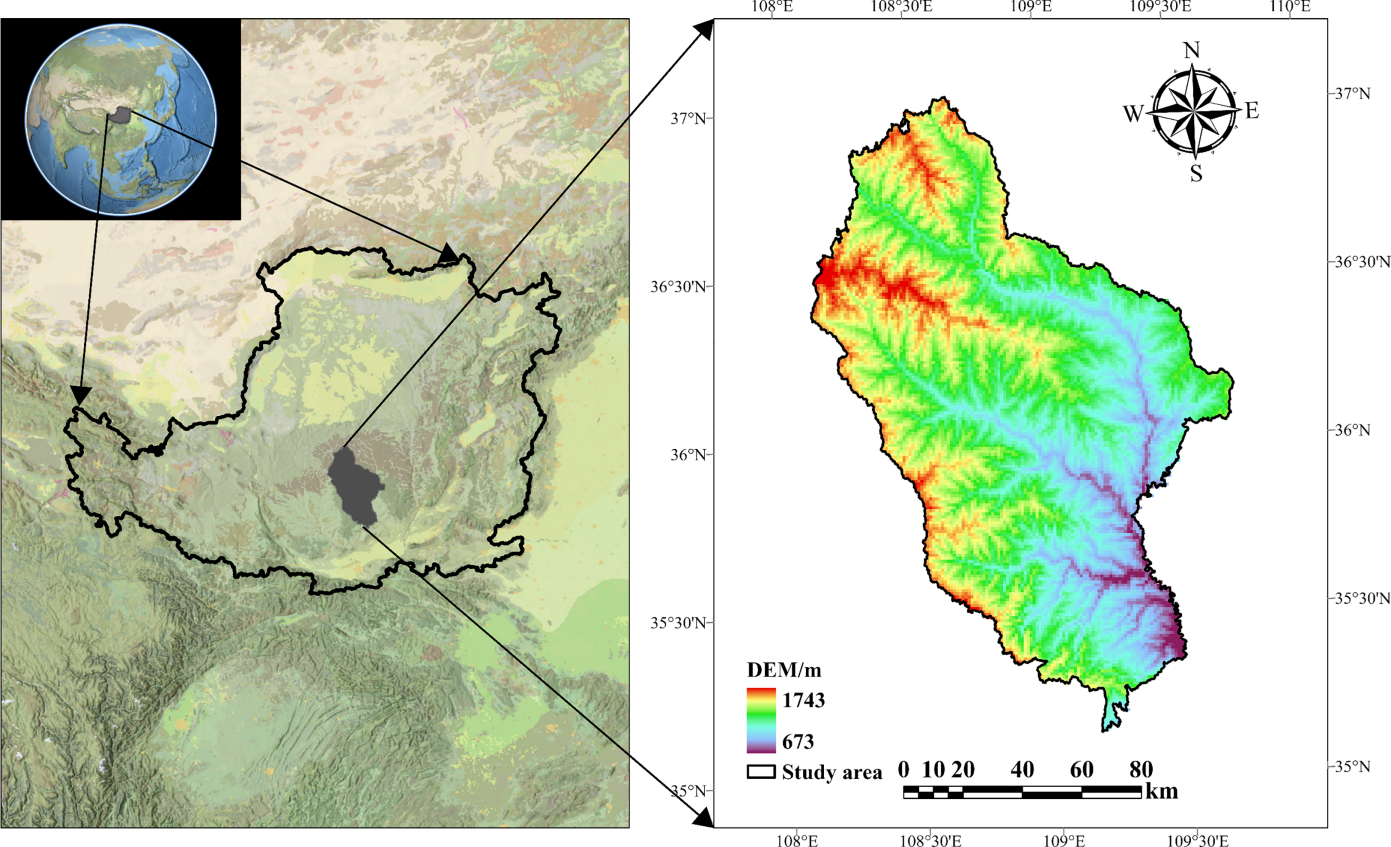
Ziwuling study area on the Chinese Loess Plateau.

The Ziwuling Nature Reserve is located in China’s temperate zone on the Loess Plateau (34°50′–36°50′N, 107°30′–109°40′E) (**Figure 1**). This reserve represents the best-preserved natural vegetation area on the Loess Plateau, with a representative plant germplasm resource base and the most important secondary primary forest (Guo and Wang, 2005). The vegetation on the ground is mainly represented by a temperate deciduous broadleaf forest dominated by *Quercus mongolica* and a temperate coniferous forest dominated by *Pinus oleifera* (Cheng et al., 2012). Zonal soil in the mountains is either primary or secondary loess with a pH of 7.5-8.2, and the soil profile is shallow overall (Tian et al., 2022).

### 2.2 Experimental design

The “space-for-time” replacement method has been widely utilized by ecologists to anticipate vegetation chronosequence change since 1899 (Cowles, 1899; Blois et al., 2013). In the present study, we used a “space for time” strategy to analyze 48 plots. From 2020 to 2021, standard sample plots of typical vegetation types were established with basically equal stand conditions and similar soil texture, with six duplicates for each restoration stage (**Table 1**). The restoration phase was determined by the findings of the relevant forestry department survey and an in-depth review of all relevant reports (Zou et al., 2002; Fan et al., 2006; Deng et al., 2013; Zhao et al., 2015; Liu et al., 2020).

**Table 1:** Vegetation survey sample site information.

Restoration time (year)	Succession stage	Plots	Slope/°	Elevation/m	Representative plants
0	Farmland	6	19–22	981–987	*Viola collina*, *Agropyron cristatum*
10	Pioneer Grassland	6	24–25	1212–1215	*Bothriochloa ischaemum*, *Lespedeza bicolor*, *Artemisia chamaemelifolia*
20	Grassland	6	16–28	994–1021	*Lespedeza bicolor*, *Artemisia codonocephala*, *Agrimonia pilosa*
40	Shrub	6	10–30	1028–1169	*Sophora davidii*, *Carex lanceolata*, *Lespedeza bicolor*
70	Pioneer arbor	6	19–30	1030–1131	*Betula platyphylla*, *Lespedeza bicolor*, *Agrimonia pilosa*
120	Sub-top stage	6	23–25	1096–1122	*Pinus tabuliformis*, *Carex lanceolata*, *Lespedeza bicolor*
135	Sub-top to top transition stage	6	13–35	1160–1246	*Pinus tabuliformis*, *Quercus mongolica*, *Lespedeza bicolor*
150	Top Stage	6	16–20	1146–1240	*Quercus mongolica*, *Carex lanceolata*, *Lespedeza bicolor*

### 2.3 Vegetation investigation and sampling

The species and their abundance in each standard sample plot (random selection) were recorded for plots of 20 m × 20 m (trees), 10 m × 10 m (shrubs), and 1 m × 1 m (grasses) (**Table 1**). The species’ Latin names were verified against the Flora of China (Li, 2007) and The Plant List (http://www.plantlist.org/). Owing to the shallow soil layer in the Ziwuling area, the improvement effect of vegetation restoration on the soil is mainly concentrated in the 0–40 cm surface soil layer (Gu et al., 2019). Therefore, a root auger with an inner diameter of 7 cm was used to collect fine root (≤2 mm), and soil samples were collected from this soil layer using a root auger with four replicates for each type of soil sample. The complete harvest method was used to collect litter from 31.7 cm × 31.7 cm plots (Ravindranath and Ostwald, 2007). The bulk density of the soil was determined using the cutting ring (100 cm^3^) method. Soil water-stable aggregates were determined using the wet sieve method (Yoder, 1936), and SOC content was determined using the dichromate oxidation method (Nelson and Sommers, 1996).

### 2.4 Data processing

The total plant diversity, woody plant diversity, and herbaceous plant diversity were determined using the following equations (Simpson, 1949; Hill, 1973):


(1) Species richness index = S,

(2) $$\text{Simpson index}=1-\sum_{i=1}^{S}p_{i}2_{,}$$

(3) $$\text{ Shannon–Wiener index=-}\sum_{i=1}^{S}p_{i}\text{ln}(p_{i}),$$

(4) $$\text{Pielou index=}\frac{\text{H}}{\text{ln}(\text{S}),}$$

where *S* is the total number of plant species in the sample quadrat, *pi* is the relative abundance of plant species *i* in one quadrat, and *H* is the Shannon–Wiener index.

SOC (g/m^2^) was calculated using Equation (5):


(5) SOC storage=X×BD×T×10,

where X. is the SOC content of the soil (g/kg), BD is the density of the bulk soil (g/cm^3^), and T is the soil layer thickness (cm).

### 2.5 Statistical analyses

Prior to analysis, all data were examined for normality and homogeneity of variance; data having non-normal distribution and/or non-homogeneous variance were log- or power function transformed to meet the assumptions for statistical analysis. Regression analysis was performed to evaluate the relationships between plant diversity and restoration stage. Carbon sequestration characteristics among the different periods were compared using one-way ANOVA. If significant effects were observed using ANOVA, the least significant difference [LSD (0.05)] test was used. Pearson’s correlations were used to determine the variables that were significantly correlated with plant diversity, restoration stage, and carbon sequestration characteristics. PLS-PM was performed to further link plant diversity with carbon sequestration characteristics. PLS-PM is a type of structural equation modelling algorithm based on correlation. The concept of causality is expressed in terms of linear conditional expectations to seek the best linear prediction relationship and allow the use of latent variables to estimate complex causality or prediction models (Sanchez, 2013). The PLS-PM method was selected because it requires a small sample size, and the algorithm can optimize the prediction of dependent variables and fitting of data to a predetermined model (Hulland, 1999; Götz et al., 2010; Ali et al., 2017). Models with different structures were evaluated using the goodness-of-fit (GOF) statistics, a measure of their overall predictive power, with GOF > 0.7 considered an acceptable value (Tian et al., 2019; Zhong et al., 2020). All statistical analyses were performed using the R software (v. 4.1.1, R Core Team, 2020).

## 3 Results and analysis

### 3.1 Vegetation composition characteristics

**Figure 2:**
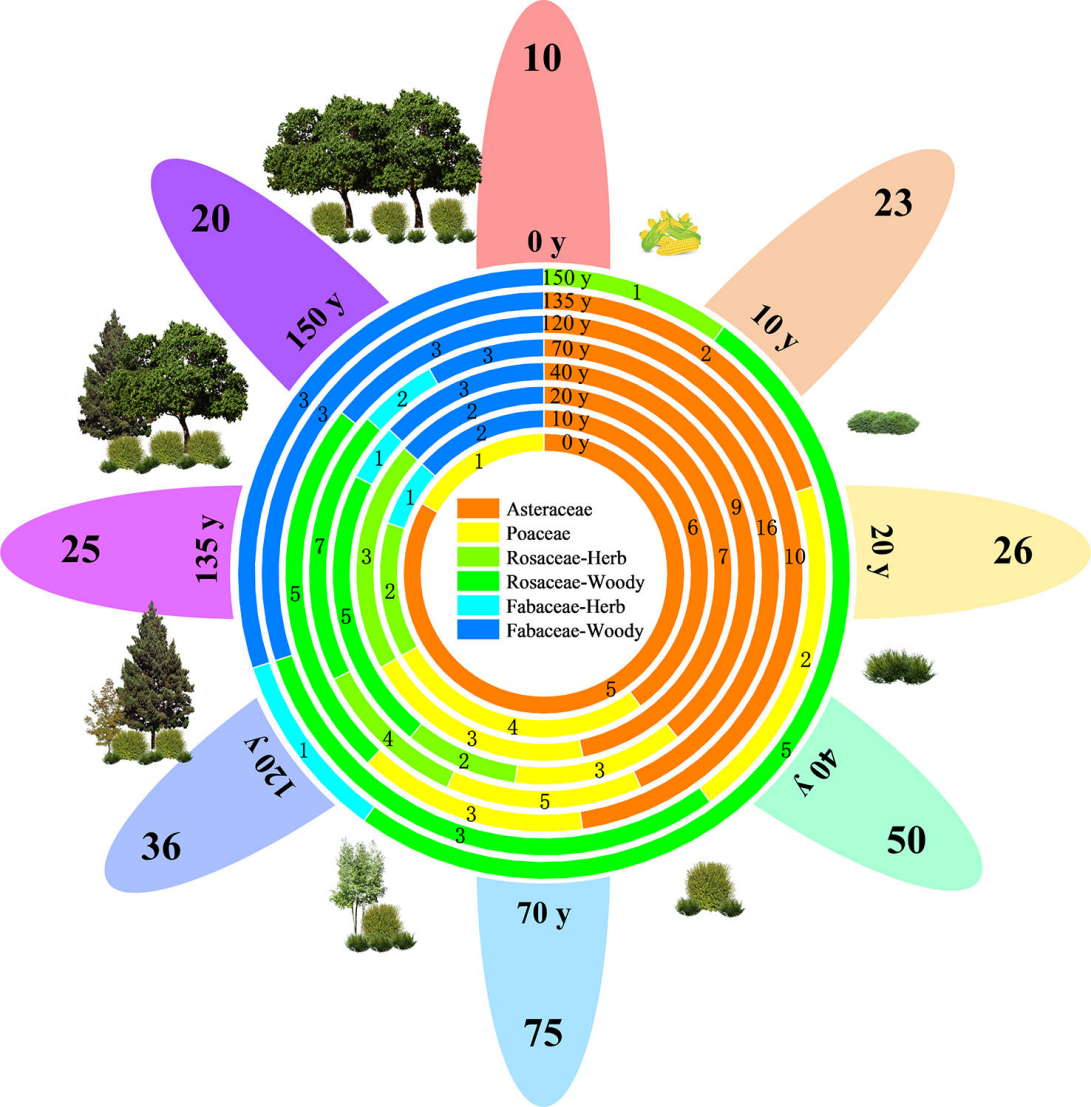
Vegetation composition and life forms of different vegetation restoration stages on the Chinese Loess Plateau. The numbers on the petal margins indicate the richness of plant species at each stage (e.g., 10). The combination containing y indicates the recovery time (e.g., 20 y). The circles in the center of the flowers indicate the species richness of different families at different stages.

**Figure 2** shows that the vegetation in the study area has been naturally restored for 150 years. A total of 128 species of seed plants were recorded in the area, belonging to 39 families and 99 genera, including two species of gymnosperms belonging to two families and two genera and 126 species of angiosperms belonging to 37 families and 97 genera. Asteraceae (17 genera and 28 species), Rosaceae (13 genera and 17 species), Poaceae (10 genera and 10 species), and Fabaceae (six genera and eight species) accounted for 49.22% of the total number of species and were the dominant families. Asteraceae and Poaceae are primarily herbaceous plants, whereas Rosaceae and Fabaceae are primarily woody plants. The plant species richness of all species and four major families showed a single-peaked curve, increasing and then decreasing with the progress of restoration, and reaching a maximum after 70 years of restoration.

### 3.2 Plant diversity characteristics

**Figure 3:**
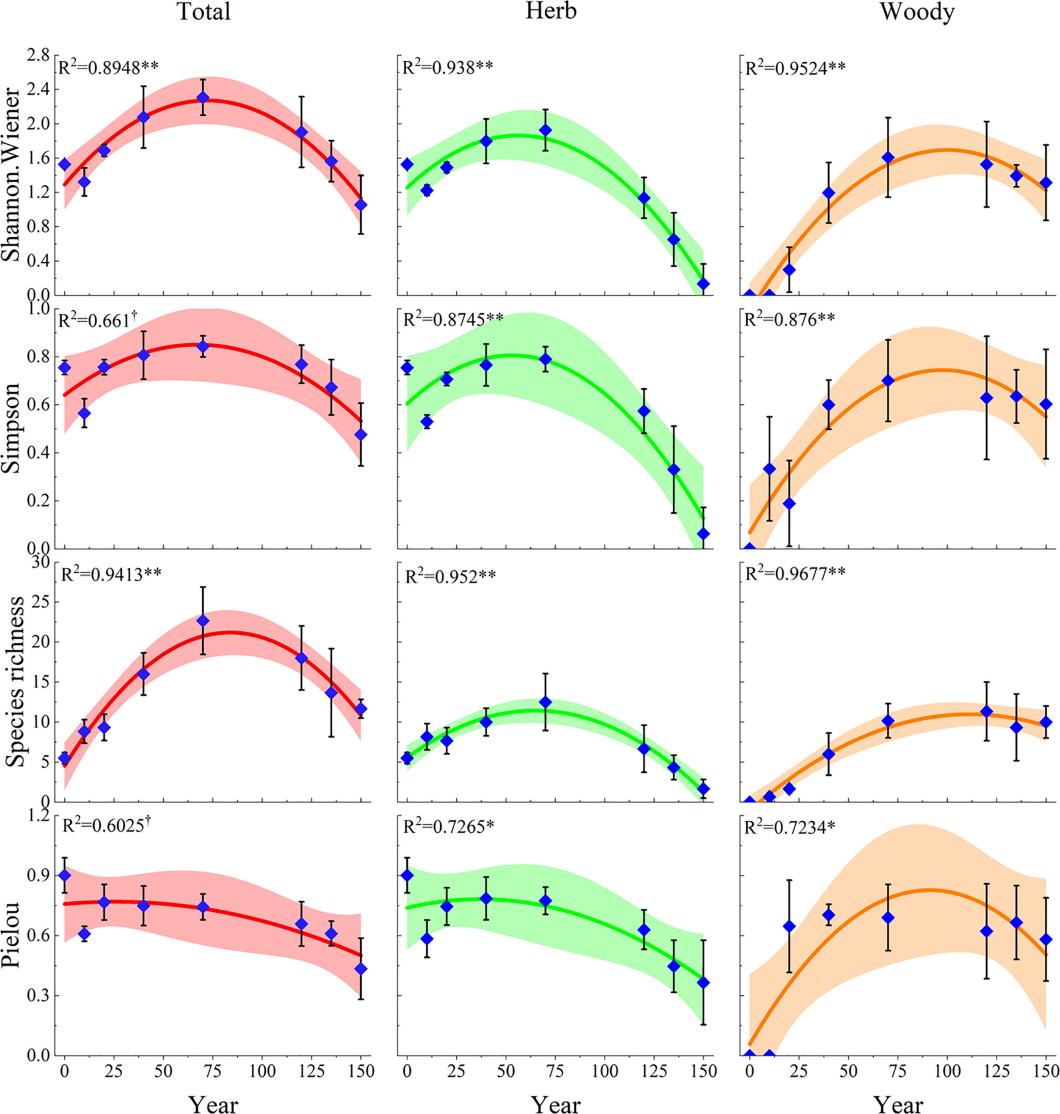
Multiple linear regression relationships of plant diversity with the natural restoration succession periods. The dark lines indicate the fits of the linear model to the plant diversity and succession ages, while the ribbon are the 95% confidence intervals of these linear models. †, *P*< 0.1; *, *P*< 0.05; **, *P*< 0.01. Total represents the overall diversity of plant communities; Herb represents the herb diversity in plant communities; Woody represents the woody plant diversity in plant communities.

The fitted curves showed that the plant diversity indices had a single-peaked curve in relation to the restoration years during the 150 years of natural vegetation restoration succession (**Figure 3**). The Species richness, Shannon-Wiener, and Simpson indices showed a similar pattern, increasing and then decreasing, with the peak of herbaceous plants occurring earlier, between 40 and 70 years of vegetation restoration, the peak of total plant diversity occurring in the middle of the restoration period, between 70 and 100 years, and the peak of woody plants occurring later, between 100 and 120 years of restoration. However, the Pielou indices of herbaceous plants and total plants showed similar trends, with their peaks occurring earlier, at approximately 20 years of natural recovery, and decreasing thereafter. The Pielou index of woody plants increased at first, peaked at approximately 90 years, and then started to decrease. This indicated that the evenness of species distribution in the community was mainly influenced by herbaceous plants. In addition, we found that herbaceous plant diversity was graphically symmetric with respect to woody plant diversity at 70 years of restoration. This reflected the differentiation of ecological niches to capture maximum possible resources.

### 3.3 Carbon sequestration characteristics

**Figure 4:**
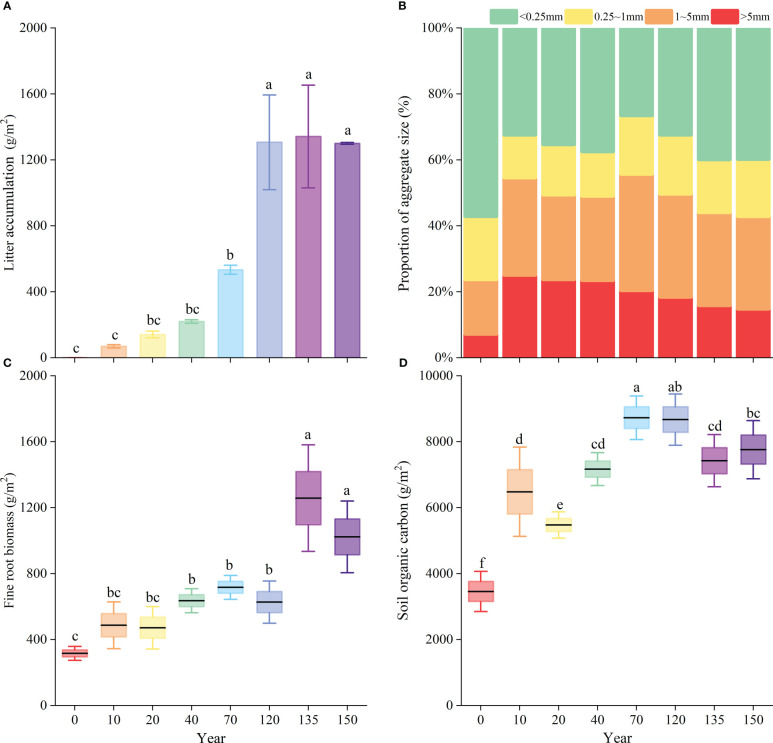
Comparison of litter **(A)**, soil aggregate **(B)**, fine-root biomass **(C)** and soil organic carbon **(D)** in each restoration succession period. Different lowercase letters (a–f) indicate significant differences (P< 0.05) within each variable among different succession periods.

**Figures 4A, C** and **D** show that with the increase in the natural vegetation recovery time, the difference in vegetation compared with the agricultural stage became more significant (*P*< 0.05). Litter accumulation, fine-root biomass, and SOC storage increased with the progress of vegetation restoration. The most considerable increase in litter accumulation was 773 g/m^2^ after 120 years of restoration compared to that after 70 years of restoration, while the differences were smaller in the later stages (**Figure 4A**). A significant increase in fine-root biomass was observed during the 120- to 135-year stage of vegetation recovery (**Figure 4C**). As the SOC storage peaked at about 9447 g/m^2^, the disparity between the agricultural stage and the 120 years of restoration stage was at its greatest (**Figure 4D**). From the agricultural stage to the 10-year natural recovery stage, the content of large-sized water-stable agglomerates increased the fastest at 24.7%, followed by 10.9% at the 40–70-year recovery stage. The >5 mm size agglomerates increased rapidly at first, peaked at 25.1% in the 10-year natural recovery stage, and then decreased gradually; the 1–5 mm size agglomerates initially increased (but fluctuated), peaked at 70 years, and then decreased gradually, while the 0.25–1 mm size agglomerates showed relatively smaller variations (**Figure 4B**).

### 3.4 Relationship between plant diversity and carbon sequestration characteristics

**Figure 5:**
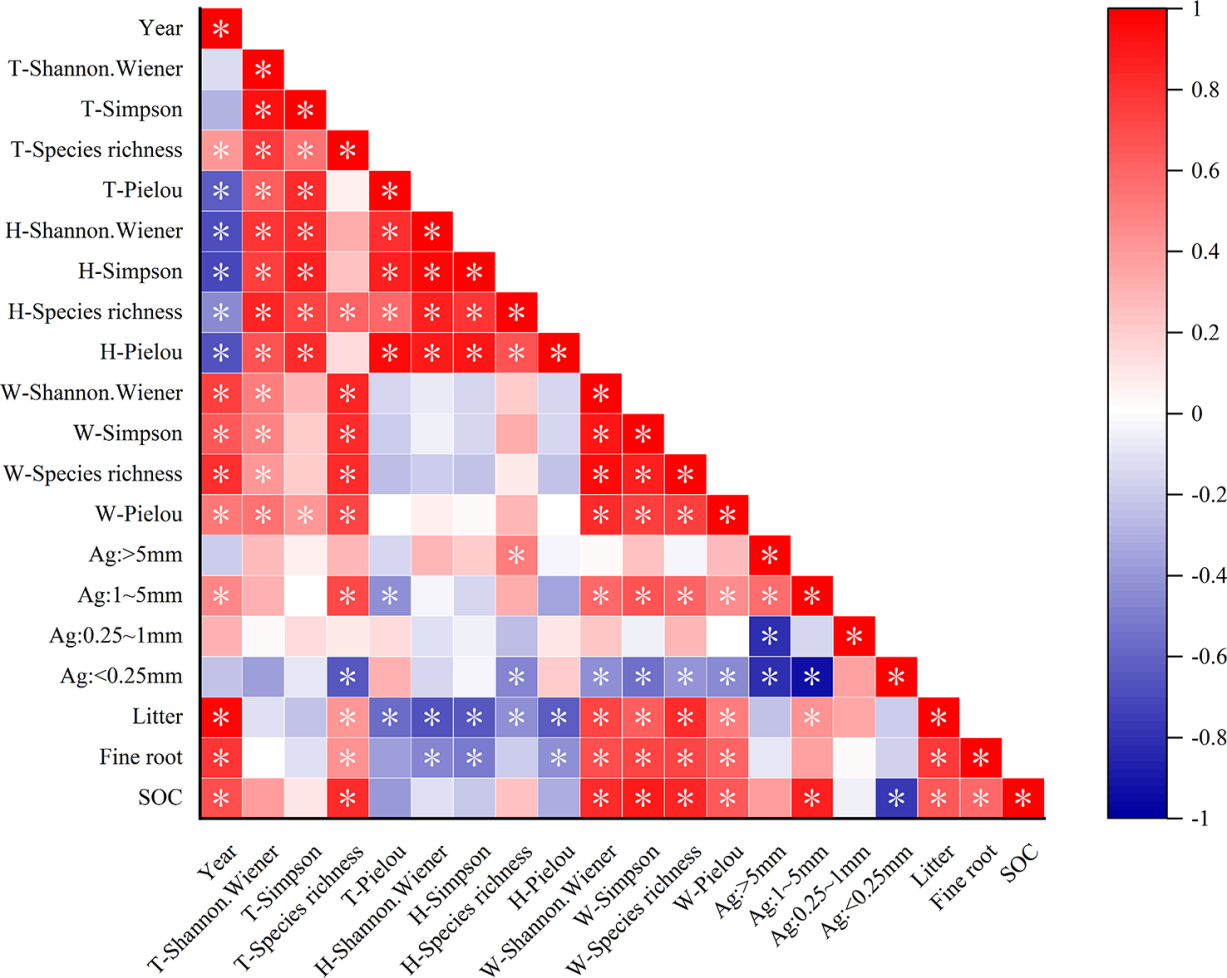
Relationship between plant diversity and carbon sequestration characteristics. *P < 0.05. T represents the overall diversity of plant communities; H represents the herb diversity in plant communities; W represents the woody plant diversity in plant communities; Ag represents the aggregate size; SOC represents the soil organic carbon. The darker the color, the stronger the correlation.

Pearson correlation analysis revealed that the total species richness, woody plant diversity index, litter, fine-root biomass, and SOC were significantly positively correlated with recovery time, whereas the herb diversity index and total Pielou index were significantly negatively correlated with recovery time (**Figure 5**). The total species richness of the plant communities was strongly positively correlated with woody plant diversity. Herb diversity was significantly positively correlated with the total Pielou, Simpson, and Shannon–Wiener indexes of the plant community. This indicated that the species variety of the entire community was controlled by woody plants, and the species number was influenced by herbaceous plants. In addition, herbaceous plant diversity was negatively correlated with fine-root biomass and litter accumulation. Woody plant diversity significantly affected the SOC storage and was positively correlated with fine-root biomass and litter accumulation, as well as with 1–5 mm grain size aggregates, which had multiple functions. Litter and fine roots were significantly positively correlated with SOC (*P<* 0.05). At the same time, agglomerates of 1–5 mm particle size were significantly positively correlated with SOC. This indicated that litter and fine roots are important for SOC accumulation, and 1–5 mm agglomerates are important carriers of SOC.

### 3.5 Plant diversity drives soil carbon sequestration

**Figure 6:**
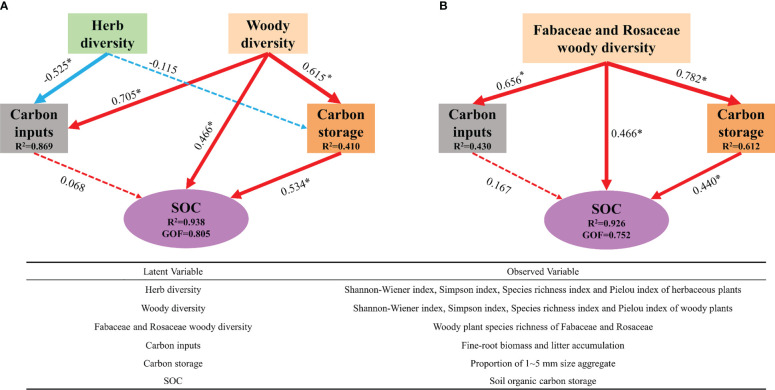
**(A)** Plant diversity drives soil carbon sequestration. **(B)** Carbon capturing plants drive soil carbon sequestration. *P < 0.05. Numbers on arrows are path coefficients indicating a positive (positive number) or negative effect (negative number) effect. The red lines represent positive effects, and blue lines represent negative effects. Solid lines represent significant effects, while dashed lines represent nonsignificant effects. The width of each arrow is proportional to the standardized path coefficients.

PLS-PM revealed a significant association between plant diversity and soil carbon sequestration-related pathways involving carbon inputs, carbon storage, and a direct path (**Figure 6A**). It explained 93.8% of the SOC sequestration variance and provided the best fit to our data (GOF = 0.805). Woody plant diversity showed the greatest positive effect on soil carbon sequestration via direct and indirect effects, whereas herb diversity was a negative determinant of the effects of carbon inputs on natural restoration succession periods. Based on our findings (**Figure 6A**), we performed a PLS-PM analysis using the number of woody Fabaceae and Rosaceae species at different recovery periods (**Figure 6B**). PLS-PM explained 92.6% of the SOC sequestration variance and provided the best fit to our data (GOF = 0.752). The results showed that Fabaceae and Rosaceae diversity, through direct and indirect effects of improving soil aggregation, significantly affected the SOC accumulation. This indicated the presence of carbon sequestration plants in these two families, which are the key species related to SOC accumulation during natural vegetation recovery.

## 4 Discussion

### 4.1 Plant diversity drives soil carbon sequestration

The present study confirmed the consistency of plant diversity recovery and soil carbon accumulation, which is consistent with the findings of several previous studies (Chen et al., 2018; Madrigal-González et al., 2020; Osuri et al., 2020; Furey and Tilman, 2021; Suryaningrum et al., 2021). However, it is important to note that soil carbon sequestration was mainly driven by woody plant diversity during the 150 years of natural recovery of temperate vegetation. This indicated the significant role of forests in carbon sequestration, not only in the ground but also in carbon input to the soil, a view that is widely shared (Besnard et al., 2021; Ding et al., 2021). As shown by the direct relationship in PLS-PM (**Figure 6A**), SOC accumulation was mediated by woody plant diversity through increased proportions of large soil aggregates and other hitherto unidentified mechanisms. The mechanisms that have been identified so far include, first and foremost, that communities with high plant diversity have higher litter accumulation, and the input of litter increases future soil carbon (Gregorich et al., 2017). Second, the input of root secretions acts as a source of soil carbon (Rasse et al., 2005; Kramer et al., 2010; Wang et al., 2021). Simultaneously, changes in deadfall and root systems can alter the activity and composition of microbial communities and affect microbial carbon turnover and carbon sequestration rates (Bais et al., 2006; Craig et al., 2022). Third, soil microbial metabolic activity can influence anabolic changes in plant diversity, which in turn affects the carbon input from vegetation to the soil (Lange et al., 2015; Manral et al., 2022; Padalia et al., 2022; Manral et al., 2023). In addition, herbaceous plant diversity decreased with increasing time of revegetation and showed a highly significant negative correlation with carbon input, suggesting that dominant populations established by herbaceous plants through competitive exclusion had a positive effect on litter and fine roots. Herbaceous plants may also influence SOC accumulation through the three pathways described above (Yang et al., 2019b). In summary, vegetation plays a significant role in the formation of soil organic matter and influences fundamental soil forming processes such as aggregation or podzolization (Awasthi et al., 2022b; Awasthi et al., 2022c). Notably, soil aggregates are known to be the main sites of SOC fixation (Shankar and Suresha, 2006; Bai et al., 2020). It is hypothesized that approximately 90% of SOC in the surface soil of terrestrial ecosystems is fixed in soil aggregates (Six and Paustian, 2014). Soil fixation of organic carbon depends on soil agglomeration (Oades and Waters, 1991), and the physical segregation of soil aggregates, which provides them a good C sequestration capacity, can slow down the rate of organic carbon loss (Six et al., 2004; De Gryze et al., 2006). Moreover, increase in the proportion of large-size aggregates due to woody plant diversity enhances the SOC storage capacity.

In the present study, the carbon input pathway for SOC accumulation in PLS-PM was not significant, probably because the entry of litter into the soil requires microbial mediation. Fine roots are mostly living roots, and their main role is exchanging material with the soil, during which they produce carbon input as biological residues, thereby affecting future soil carbon (Gregorich et al., 2017). However, in the present study, Pearson’s correlation analysis found that both deadfall and fine roots increased significantly with the natural vegetation recovery process, and SOC accumulated gradually (**Figures 4**, **5**). In summary, from our observations of 150 years of natural vegetation recovery succession, we hypothesized that the positive effect of plant diversity on carbon sequestration was driven by the input of carbon into the soil by litter and roots that was stored in large particle size aggregates, in which soil microorganisms play an important role. This mechanism is more evident in woody plants than in other plants. Some evidence suggests that our proposed mechanism is of general interest (Prommer et al., 2020; Jia et al., 2021; Wang et al., 2021; Feng et al., 2022). This study emphasized that a wide variety of plants is important and can have many ecological and environmental benefits, such as greater carbon sequestration.

### 4.2 Carbon capturing plants drive soil carbon sequestration

In the present study, we found that Fabaceae and Rosaceae plant diversity is important for SOC accumulation, and that plants belonging to these two families can directly transport organic carbon to the soil through the mechanisms described previously. It should not be overlooked that most of these species are well-rooted plants, and they can increase the content of large particle size aggregates (1–5 mm) in the soil and enhance the soil carbon sequestration capacity. This inter-rooting effect can also significantly improve the stability of soil aggregates and promote carbon sequestration by soil aggregates (Li et al., 2020). An increase in nitrogen content has been shown to be highly correlated with an increase in SOC (Li et al., 2022), and an increase in SOC content under high-nitrogen conditions corresponds to a 33% reduction in CO_2_ outflow (Tian et al., 2019). Fabaceae and nitrogen-fixing bacteria are known to form specific host relationships (Dassen et al., 2017). Several carbon sequestration pathways showed that both families contained species that could efficiently sequester carbon.

We screened three key species (*L. bicolor, S. davidii*, and *C. multiflorus*) taking into account the two criteria that the plants must be woody and in either Rosaceae or Leguminosae, as well as the reported plant importance values (Tian et al., 2022). All three species are shrubs; therefore, the carbon- sequestration function of shrubs should not be neglected in the process of revegetation. All three plants grow in temperate conditions, which is consistent with the climatic characteristics of the study area. Furthermore, Tian et al. (2022) showed that the ecological niche breadth of *L. bicolor*, *S. davidii*, and *C. multiflorus* was 9.42, 4.36, and 3.34, respectively, and they easily co-occur with other species. This indicates that they are well adapted and are important for maintaining community stability and active vegetation recovery in the area. According to Kou (2016), these three species are highly erosion-resistant and have high ecological value. Overall, we concluded that reasonable allocation of shrubs in artificial vegetation restoration can improve the vegetation carbon sequestration capacity, ecological service function, and soil health. In this study area, these three species can be considered for inclusion in artificial vegetation construction. Among them, *L. bicolor* is probably the best choice because it has been found to contain a variety of effective rhizobacteria (Yao et al., 2002). Moreover, the nitrogen in its senescing leaves is rarely transferred and is fed back to the soil in the form of litter and it has a significant impact on soil nitrogen and SOC accumulation because of the appropriate rate of litter decomposition (Hendricks and Boring, 1992). It has also been found that transplantation of this species to poorly eroded soils resulted in SOC enrichment and significantly improved cluster stability (Bin and Xin-Hua, 2006; Yao et al., 2009). This evidence is consistent with the results of the present study and suggests that *L. bicolor* has great potential for improving vegetation carbon sequestration capacity and deserves further study.

## 5 Conclusions

This study confirmed that the restoration of plant diversity is consistent with soil carbon accumulation. Plant diversity showed a single-peaked curve during 150 years of vegetation restoration. The peak of herbaceous plant diversity occurred between 40 and 70 years of vegetation recovery, and the peak of woody plant diversity occurred between 70 and 120 years of vegetation recovery. Litter accumulation, fine-root biomass, and SOC storage increased with the progress of vegetation restoration. The positive effect of plant diversity on carbon sequestration may be related to litter accumulation and fine-root biomass, which drive carbon input and storage in large-particle-size (1–5 mm) aggregates. Furthermore, woody plant diversity was revealed to be the primary driver of soil carbon sequestration, whereas plant diversity in Fabaceae and Rosaceae was discovered to be crucial for SOC accumulation. The vegetation carbon sequestration function of shrubs should not be neglected during vegetation restoration. In the study area, three species, namely *L. bicolor*, *S. davidii*, and *C. multiflorus*, should be considered for plantation. Among them, *L. bicolor* was observed to be the best choice. Overall, this study provides important insights into the driving mechanisms underlying plant diversity and soil carbon sequestration and its implications for addressing the effects of global climate. To quantitatively identify the contribution of different mechanisms to soil carbon sequestration, future research needs to incorporate more biotic (e.g., microbial) and abiotic factors. This will be useful for fully clarifying the connections between the above- and belowground components of temperate vegetation.

## Data availability statement

The original contributions presented in this study are included in the article/supplementary material. Further inquiries can be directed to the corresponding author.

## Author contributions

Conception and design of study: QT, XZ. Acquisition of data: QT, XZ, HY, XX, JH, LH. Analysis and/or interpretation of data: QT, XZ, YL. Drafting the manuscript: QT. Revising the manuscript critically for important intellectual content: XZ, YL. All authors contributed to the article and approved the submitted version.
